# A Simple and Quick Method for Loading Proteins in Extracellular Vesicles

**DOI:** 10.3390/ph14040356

**Published:** 2021-04-13

**Authors:** Sara Busatto, Dalila Iannotta, Sierra A. Walker, Luisa Di Marzio, Joy Wolfram

**Affiliations:** 1 Department of Biochemistry and Molecular Biology, Mayo Clinic, Jacksonville, FL 32224, USA; iannotta.dalila@mayo.edu (D.I.); walker.sierra1@mayo.edu (S.A.W.); 2 Vascular Biology Program, Boston Children’s Hospital, Boston, MA 02115, USA; 3 Department of Surgery, Boston Children’s Hospital and Harvard Medical School, Boston, MA 02115, USA; 4 Department of Pharmacy, University of Chieti—Pescara “G. d’Annunzio”, 66100 Chieti, Italy; l.dimarzio@unich.it; 5 Department of Nanomedicine, Houston Methodist Research Institute, Houston, TX 77030, USA

**Keywords:** Cas9, drug delivery, exosome, extracellular vesicles, protein delivery

## Abstract

Extracellular vesicles (EVs) mediate intercellular transport of biomolecular cargo in the body, making them promising delivery vehicles for bioactive compounds. Genetic engineering of producer cells has enabled encapsulation of therapeutic proteins in EVs. However, genetic engineering approaches can be expensive, time-consuming, and incompatible with certain EV sources, such as human plasma and bovine milk. The goal of this study was to develop a quick, versatile, and simple method for loading proteins in EVs post-isolation. Proteins, including CRISPR associated protein 9 (Cas9), were bound to cationic lipids that were further complexed with MDA-MB-231 cell-derived EVs through passive incubation. Size-exclusion chromatography was used to remove components that were not complexed with EVs. The ability of EVs to mediate intracellular delivery of proteins was compared to conventional methods, such as electroporation and commercial protein transfection reagents. The results indicate that EVs retain native features following protein-loading and obtain similar levels of intracellular protein delivery as conventional methods, but display less toxicity. This method opens up opportunities for rapid exploration of EVs for protein delivery.

## 1. Introduction

In recent decades, extracellular vesicles (EVs) have generated considerable interest due to their involvement in physiological and pathological intercellular communication [1,2,3,4]. EVs are composed of an external bilayer formed by lipids [5], proteins [6], and glycans [7] that enclose an aqueous core with additional bimolecular cargo, such as nucleic acids [8]. EVs are released by all cells and are classified based on biogenesis, but subtypes also differ in terms of size and composition. Exosomes are small EVs (approximately 30–100 nm) that are derived from multivesicular bodies that fuse with the cell membrane [2,9]. Microvesicles are larger EVs (approximately 100–1000 nm) that are formed through cell membrane budding [2]. Apoptotic bodies are the largest EV subtype (approximately 1000–4000 nm) and are formed during apoptosis through blebbing of the cell membrane [10,11]. Exosomes, microvesicles, and apoptotic bodies have overlapping size and biomolecular cargo, making it challenging to isolate and identify specific EV types [12]. 

The inherent ability of EVs to carry biomolecular cargo makes them promising drug delivery systems [13,14,15]. In fact, synthetic liposomes, which are structurally similar to EVs, have been used in clinical applications as drug carriers for decades [16,17]. Encapsulation of therapeutic agents in nanodelivery systems can decrease renal clearance, improve accumulation in target tissue(s), and reduce toxicity [18,19,20,21]. Additionally, drug delivery carriers play an important role in protecting therapeutic agents from enzymatic degradation in circulation and the extracellular space, as well as facilitating drug solubilization, intracellular uptake, and combination therapy [22,23,24,25]. Over 50 nanomedicines have received clinical approval for various diseases due to improved efficacy and/or safety [26]. However, these nanoparticles have few components and are based on simple structures and designs that perform only marginally better than free drug counterparts. Manufacturing challenges often limit the development of complex drug delivery systems with numerous components, thereby restricting functional performance. For example, clinically approved liposomes have up to four lipid components and lack proteins and glycans, which is in stark contrast to EVs that have hundreds of different biomolecules. The complex composition of EVs is a result of millions of years of evolutionary selection for effective biomolecular transport and/or intercellular communication. It is likely that the complex and multipronged nature of EVs will render them superior to synthetic drug carriers in overcoming different biological hurdles to obtain optimal extracellular and intracellular drug delivery. For example, a recent mouse study demonstrated that EVs were more efficient at delivering functional small interfering RNA (siRNA) than synthetic lipid nanoparticles [27].

Compared to small molecules, protein-based therapeutic agents are larger and more sensitive to degradation, making delivery more challenging. Cationic lipids and polymers have been shown to form complexes with RNA and proteins, facilitating intracellular delivery, and endosomal escape [28,29,30]. However, cationic nanoparticles are often toxic, causing immune activation, vacuolation of the cytoplasm, and cell shrinkage [31,32,33,34], necessitating the development of alternative approaches for protein delivery. Several methods have been developed for loading therapeutic proteins into EVs, which include genetic engineering of producer cells [35]. For example, a mutant form of the anti-apoptotic protein survivin was expressed in melanoma cells, causing them to release EVs containing the protein. The EVs had anti-cancer properties in pancreatic cancer cells as the encapsulated mutant protein blocked the effects of survivin, triggering apoptosis [36]. Other studies have loaded antigens [37] or CRISPR associated protein 9 (Cas9) into EVs through genetic engineering of producer cells [38,39,40]. However, genetic engineering approaches, which can be expensive and time-consuming, can only be used with EVs obtained from cell culture. It is necessary to use other methods to load therapeutic agents into EVs obtained directly from human plasma [41,42,43,44], adipose tissue [45,46,47], and breast milk [48,49,50]. Additionally, it may be challenging or impossible to implement genetic engineering approaches for EVs obtained from plants [51,52] and animal sources, such as bovine milk [53,54,55,56,57]. Sources such as plasma and lipoaspirate are readily available and easily accessible, making them suitable for cost-effective, time-efficient, scalable, and high-yield EV isolation [58]. Previously, it has been shown that a heterogenous mixture of adipose tissue-derived EVs could be efficiently loaded with therapeutic agents [45]. It remains unknown whether further purification of EVs into distinct subtypes would improve drug loading capabilities without substantially complicating scalable manufacturing. 

Several methods have been developed for drug loading post-EV isolation, including mixing, electroporation, sonication, freeze-thaw cycles, extrusion, and the use of pore-forming agents [35]. In the case of protein loading, sonication and extrusion were found to result in superior loading efficiencies compared to mixing, freeze/thaw cycles and pore-forming agents [59]. However, these methods can permanently damage the EV membrane and cause aggregation of the therapeutic agent, thereby preventing encapsulation in EVs [27,35,60]. In fact, aggregation of therapeutic agents may cause false estimates of drug loading, as aggregates are resistant to enzymatic digestion and are challenging to separate from EVs due to overlapping size [27,60]. Additionally, it has been shown that electroporation suppresses the effects of endogenous microRNAs in EVs, indicating that the protective function of EVs is compromised by this loading method [27]. For these reasons it is important to develop new drug loading methods that do not damage EVs and increase drug delivery efficiency.

An alternative approach based on the incorporation of synthetic nanoparticles with EVs has been used for DNA [61] and RNA [62] loading. For example, the fusion of plasmid-containing liposomes with EVs enabled efficient transfection of mesenchymal stem cells, which did not occur with liposomes alone [61]. However, these hybrid approaches have not yet been reported for protein loading into EVs. This study describes a simple, versatile and quick protein loading method based on a hybrid system that incorporates lipid nanoparticles composed of di-octadecyl-amido-glycyl-spermine (DOGS; PULSin [29,30,63,64]). DOGS is a cationic lipid that binds to proteins to form synthetic lipid nanoparticles, which in this study, are shown to form a complex with EVs under optimized conditions. 

## 2. Results

### 2.1. EVs Retain Characteristic Features Pre- and Post-Loading of Cas9

**Figure 1:**
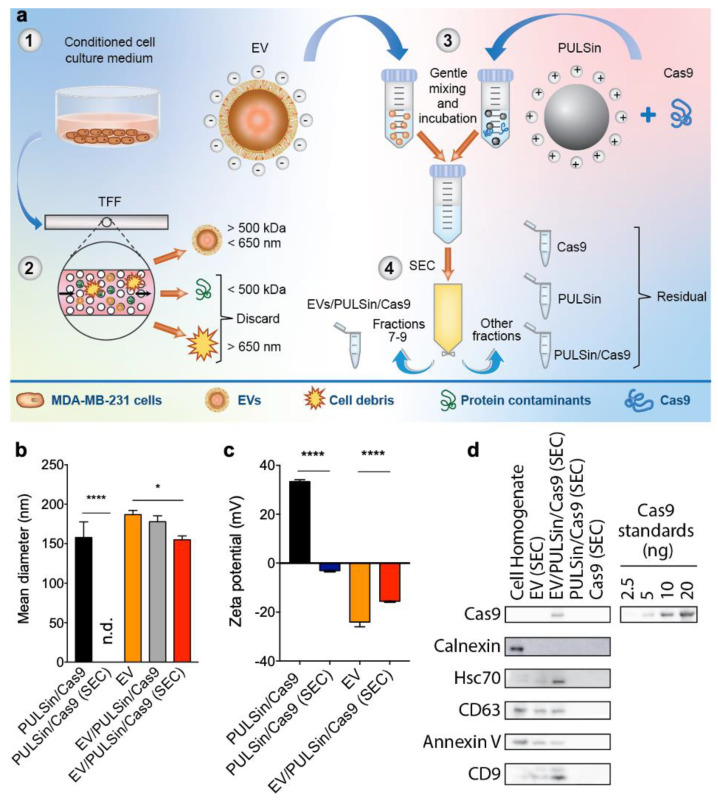
Characterization of extracellular vesicles (EVs) loaded with CRISPR associated protein 9 (Cas9). (**a**) Schematic of loading procedure. The conditioned medium of MDA-MB-231 human breast cancer cells was collected (step 1) and tangential flow filtration (TFF) was performed to remove components larger than 650 nm and smaller than 500 kDa (step 2). The isolated EVs were gently mixed with PULSin/Cas9 (step 3) and size-exclusion chromatography (SEC) was used to remove free Cas9, PULSin and Cas9/PULSin (step 4). (**b**) Mean size determined by nanoparticle tracking analysis (NTA). (**c**) Zeta potential measured by laser Doppler micro-electrophoresis. (**d**) Cas9 loading and protein markers of EVs, heat-shock cognate protein 71 (Hsc70), cluster of differentiation (CD)63, CD9 and annexin V, and intracellular contaminant marker, calnexin, detected by Western blot. Data are presented as mean ± SD of three biological replicates (b and c). Statistical analysis was performed by one-way analysis of variance (ANOVA) with post-hoc pairwise comparisons using Tukey’s test. *, *p* < 0.05; ****, *p* < 0.0001.

The goal of this study was to develop a simple, versatile and quick method for loading proteins into EVs post-isolation. EVs were isolated from the conditioned medium of human MDA-MB-231 breast cancer cells cultured for 48 h in EV-depleted medium. EVs were separated from other medium components through tangential flow filtration. Consistent with previously published studies [45,46,65], tangential flow filtration resulted in separation and concentration of nanosized EVs (Figure 1b, Supplementary Figure S1) that exhibited a negative zeta potential as expected (Figure 1c). Isolated EVs contained Hsc70, annexin V, CD63, and CD9 (Figure 1d), which are characteristic markers [12] and were negative for calnexin, an endoplasmic reticulum protein (Figure 1d) typically used as an indicator of intracellular vesicle contaminants [12]. 

For protein loading studies, Cas9 was selected as a model protein due to the importance of this enzyme in genome editing applications [66]. Cas9 was mixed with PULSin to generate synthetic cationic nanoparticles that complexed with EVs through charge-based interactions. The mixed samples were then processed through a size-exclusion chromatography column to separate EVs from excess PULSin, Cas9 and PULSin/Cas9. PULSin/Cas9 nanoparticles displayed a mean diameter of 158 nm (Figure 1b) and were not eluted in the same fractions as EVs following size-exclusion chromatography, as evidenced from the absence of any detectable nanoparticles (Figure 1b), a lack of a characteristic positive zeta potential (Figure 1c) and undetectable Cas9 (Figure 1d). These results indicate that PULSin/Cas9 does not elute in the EV fraction following size-exclusion chromatography despite having a similar size as EVs. This is likely due to the cationic charge of PULSin/Cas9, which impacts the interactions with the resin of the size-exclusion chromatography column. Protein-loaded EVs showed a clear CD63 and annexin V signal similar to that of unloaded EVs. There was an increase in CD9 and Hsc70 detected in the protein-loaded EVs (Figure 1d), which may be due to changes in interactions with the resin of the size-exclusion chromatography column following protein loading. Quantification of the Cas9 positive band in the EV/PULSin/Cas9 sample purified by size-exclusion chromatography demonstrated that between 5 ng and 10 ng of Cas9 was complexed with 3.5 × 10^8^ EVs (Figure 1d). A densitometry-based standard curve from the Western blot demonstrated that 7.7 ng of Cas9 was loaded in 3.5 × 10^8^ EVs. PULSin/Cas9 displayed a positive zeta potential of ~38 mV (Figure 1c), which is expected for cationic nanoparticles. The zeta potential of EVs increased slightly from ~−25 mV to ~−18 mV upon complexation with PULSin/Cas9 (Figure 1c). It is critical that EVs retain a negative zeta potential following protein loading, as cationic nanoparticles are known to elicit toxic effects in vitro and in vivo [31,32]. Taken together, EV properties such as overall size distribution and CD63 and annexin V expression remained unchanged upon passive incubation with PULSin/Cas9 followed by size-exclusion chromatography, while there was a slight increase in mean size and zeta potential.

### 2.2. EV-Mediated Protein Delivery 

**Figure 2:**
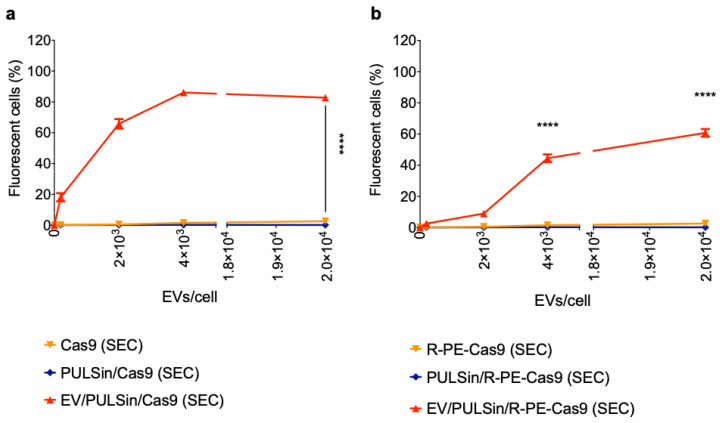
Uptake of Cas9 and Cas9-R-phycoerythrin (R-PE)-loaded EVs in Raw 264.7 macrophage cells. MDA-MB-231 human breast cancer cell-derived EVs were loaded with PULSin/Cas9 (Cas9 was labeled with Alexa Fluor 488) or PULSin/Cas9-R-PE. SEC was used to remove free protein, PULSin and PULSin/protein. The Cas9 or Cas9-R-PE amount in each sample loaded through SEC was equivalent and cells were treated with an equal volume of the elution fraction 7–9. Cellular uptake was assessed by flow cytometry after a three-hour incubation period. (**a**) Uptake of Cas9-loaded EVs and controls. (**b**) Uptake of R-PE-Cas9-loaded EVs and controls. Data are presented as mean ± SD of three replicates. Statistical analysis was performed by two-way ANOVA with post-hoc pairwise comparisons using Tukey’s test. ****, *p* < 0.0001 in comparison to PULSin/Cas9 (SEC) or Cas9 (SEC). In figure a, **** pertains to all doses except 0 EVs/cell.

**Figure 3:**
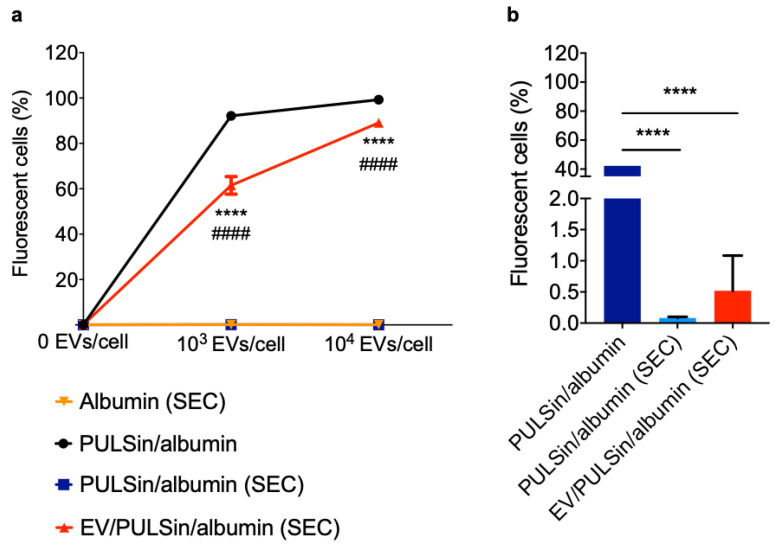
Uptake of albumin-loaded EVs in Raw 264.7 macrophages and MDA-MB-231 breast cancer cells. MDA-MB-231 human breast cancer cell-derived EVs were loaded with PULSin/albumin (albumin was labeled with Alexa Fluor 488). SEC was used to remove free albumin, PULSin and PULSin/albumin. The albumin amount in each sample loaded through SEC was equivalent and cells were treated with an equal volume of the elution fraction 7–9. Cellular uptake was assessed by flow cytometry after a three-hour incubation period. a Uptake of albumin-loaded EVs and controls in Raw 2647 cells. b Uptake of albumin-loaded EVs (10^3^ EVs/cell) and controls in MDA-MB-231 cells. Data are presented as mean ± SD of three replicates. Statistical analysis was performed by two-way (**a**) or one-way ANOVA (**b**) with post-hoc pairwise comparisons using Tukey’s test. ****, *p* < 0.0001 in comparison to PULSin/albumin SEC; ####, *p* < 0.0001 in comparison to albumin (SEC) or PULSin/albumin (SEC).

Cas9 was labeled with Alexa Fluor 488 (0.547 kDa [67]) in order to enable fluorescent quantification of cellular uptake in Raw 264.7 macrophages. Flow cytometry revealed that Cas9-loaded EVs were efficiently taken up by cells after a three-hour incubation period (Figure 2a). The uptake was dose-dependent and reached a maximum plateau at a dose of 4 × 10^3^ EVs/cell (Figure 2a). At the highest dose assessed (2 × 10^4^ EVs/cell), ~83% (±4% SD) of cells were positive for Cas9-Alexa Fluor 488. To assess the versatility of the EV-based protein delivery method, smaller and larger protein constructs than Cas9 (160 kDa [68]) were evaluated. Cas9 was conjugated to the fluorescent protein, R-PE (~240 kDa [69]), to obtain a larger construct (~400 kDa) that could be detected through flow cytometry. The results revealed that EV-based delivery of Cas9-R-PE was successful, but less efficient than Cas9 alone (Figure 2b), indicating that higher molecular weights have lower delivery efficiencies with this protocol. Specifically, at a dose of 2 × 10^2^ EVs/cell and 2 × 10^3^ EVs/cell, the uptake of Cas9 was approximately seven times higher compared to Cas9-R-PE (Figure 2). At the highest dose assessed (2 × 10^4^ EVs/cell) ~65% (±4% SD) of cells were positive for Cas9-R-PE (Figure 2b). To assess the ability of this platform to deliver smaller proteins, fluorescent albumin (66 kDa) was complexed with EVs. At the highest dose assessed (2 × 10^4^ EVs/cell) ~89% (±1.6% SD) of cells were positive for fluorescent albumin (Figure 3a), which was slightly higher than that of Cas9 (Figure 2a). The EV-mediated delivery efficiency was also compared to that of the cationic PULSin nanoparticle, which resulted in slightly higher uptake of albumin in Raw 265.7 cells—that is, ~99% (±0.8% SD) at the maximum dose (Figure 3a). 

Macrophages are known to have much higher levels of endocytosis compared to other cell lines, such as MDA-MB-231 breast cancer cells [70]. It is expected that EVs would be taken up in higher amounts by Raw 264.7 macrophages than MDA-MB-231 breast cancer cells, as endocytosis is the main mechanism by which EVs are internalized [71]. To assess whether this was the case, MDA-MB-231 cells were incubated with fluorescent albumin-loaded EVs for three hours. The results indicate that uptake was negligible in this non-macrophage cell line (~0.5% of cells were positive for fluorescent albumin at a dose of 10^3^ EVs/cell) (Figure 3b). The uptake efficiency is in stark contrast to that of the cationic PULSin nanoparticle, which resulted in approximately 40% of MDA-MB-231 cells being positive for fluorescent albumin (Figure 3b). The higher levels of uptake seen with cationic nanoparticles suggest that uptake may be taking place through non-endocytosis mediated mechanisms, which has previously been reported for cationic nanoparticles [72,73]. The low levels of EV-mediated uptake in a non-macrophage cell line, does not necessarily limit the applicability of this system and suggest that longer incubation times may be required for non-macrophage cell culture studies. Additionally, in vivo studies have demonstrated that intravenously injected EVs are primarily taken up by hepatic macrophages, making the liver an ideal organ for EV-based therapies [74,75]. However, this protein loading method could be used with engineered EVs or optimal EV subpopulations to avoid immunological clearance and improve site-specific delivery [35]. 

### 2.3. EV-Mediated Protein Uptake Is Comparable to Conventional Methods

**Figure 4:**
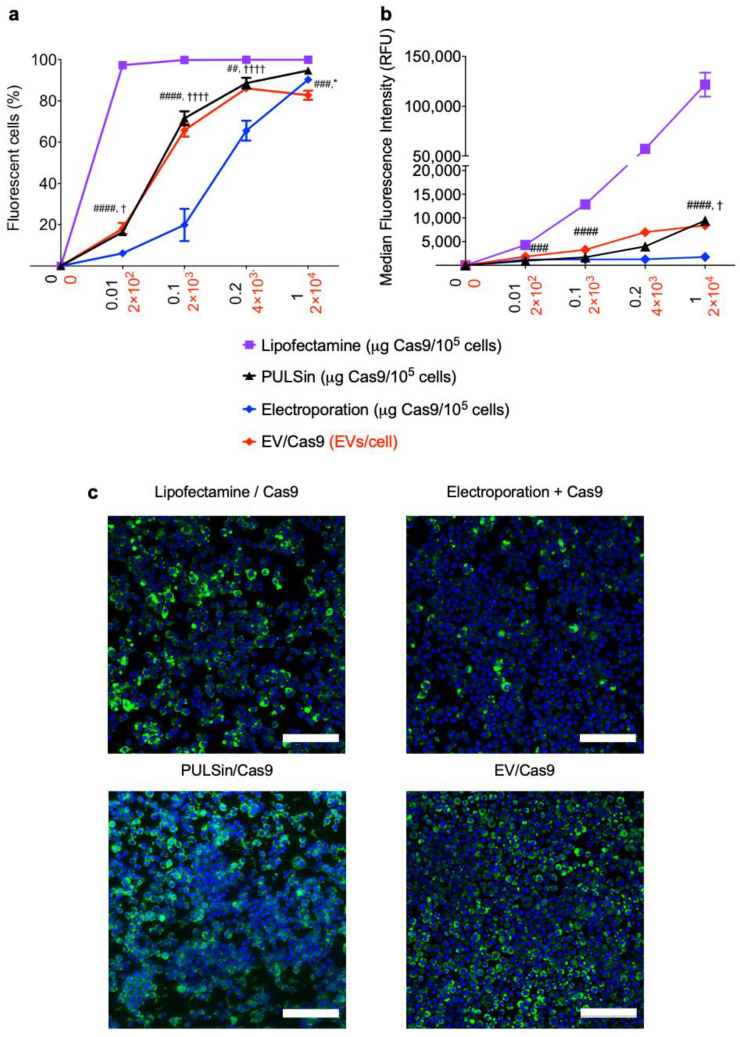
Comparison of EVs, electroporation and commercial protein transfection reagents for intracellular delivery of Cas9. Alexa Fluor 488-labeled Cas9 uptake in Raw 264.7 cells treated with commercial protein transfection reagents (PULSin or Lipofectamine CRISPRMAX Cas9), electroporation and EVs. (**a**) Percentage of fluorescent cells assessed by flow cytometry. (**b**) Median fluorescent intensity expressed in relative fluorescent units (RFU) assessed by flow cytometry. Measurements units for the x-axes are indicated in parenthesis after the sample name (black values indicate units for lipofectamine, PULSin and electroporation, while red values indicate units for EVs). Data in figure a and b are presented as mean ± SD of three replicates. (**c**) Confocal fluorescent microscopy images. Scale bars, 100 µm. Statistical analysis was performed by two-way ANOVA with post-hoc pairwise comparisons using Tukey’s test. *, *p* < 0.05 in comparison to PULSin/Cas9. ##, *p* < 0.01; ###, *p* < 0.001; ####, *p* < 0.0001 in comparison to Lipofectamine/Cas9. †, *p* < 0.05; ††††, *p* < 0.0001 in comparison to electroporated Cas9.

Many protein-delivery methods have been developed for in vitro and/or in vivo applications, including synthetic nanoparticles [29] and physical methods, such as electroporation [76]. In this study, the efficiency of EV-mediated protein delivery was compared to that of conventional methods, including commercial protein transfection reagents (PULSin and Lipofectamine CRISPRMAX Cas9) and electroporation. EVs loaded with Cas9-Alexa Fluor 488 outperformed electroporation in Raw264.7 macrophages and resulted in similar uptake levels as PULSin-mediated delivery both in terms of the percentage of fluorescent cells (Figure 4a) and median fluorescence intensity (Figure 4b). Lipofectamine was the most efficient at mediating intracellular uptake of Cas9 (Figure 4a,b). At the highest doses of Cas9 (1 µg/10^5^ cells) and EVs (2 × 10^4^/cell) all four methods resulted in over 78% of cell being positive for Cas9 (Figure 4a). Next, uptake of fluorescent Cas9 was visualized with confocal fluorescent microscopy. Cas9-Alexa Fluor 488 was visible in all treatment groups, with the electroporated cells showing the lowest signal (Figure 4c). Notably, the cells treated with EVs were more confluent than the other groups (Figure 4c). Overall, flow cytometry and fluorescent microscopy results indicate that EVs deliver Cas9 to Raw 264.7 cells with similar efficiency as commercial protein transfection reagents, while outperforming electroporation. 

### 2.4. Loaded EVs Have a Negligible Impact on Cell Viability Compared to PULSin 

**Figure 5:**
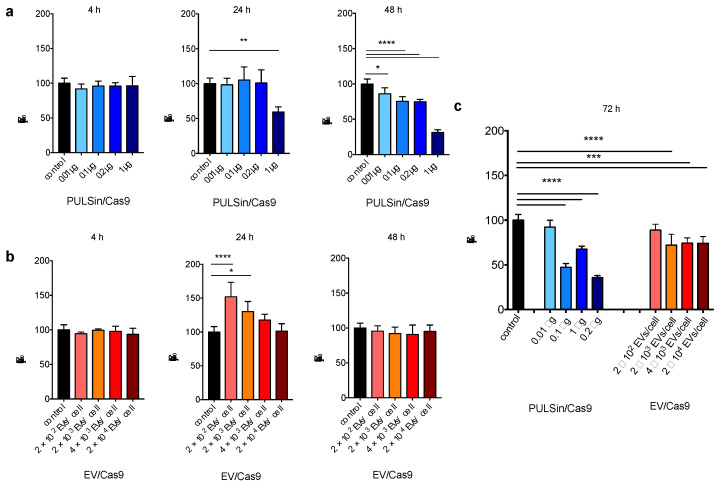
Viability of RAW 264.7 cells exposed to Cas9-loaded PULSin or Cas9-loaded EVs. (**a**) Cells treated with Cas9-loaded PULSin 4, 24, and 48 h. (**b**) Cells treated with Cas9-loaded EVs 4, 24, and 48 h. (**c**) Cells treated with Cas9-loaded PULSin or Cas9-loaded EVs for 72 h. Data are normalized to untreated control cells (data is shown twice in a and b for comparison purposes) and presented as mean ± SD of five replicates. Statistical analysis was performed by one-way ANOVA with post-hoc pairwise comparisons using Tukey’s test. *, *p* < 0.05; **, *p* < 0.01; ***, *p* < 0.001; ****, *p* < 0.0001.

Although conventional protein delivery techniques display high efficiencies, they often cause cell damage [33,34,77]. For example, cationic nanoparticles can induce cell shrinkage, and vacuolation of the cytoplasm, leading to reduced cell viability [31,32,33,34]. On the contrary, preclinical and clinical EV studies have demonstrated negligible cytotoxicity and limited immunogenicity [13,35,78,79]. In this study, the viability of Raw264.7 cells in response to Cas9-loaded PULSin (cationic nanoparticle) and Cas9-loaded EVs was compared. The results indicate that PULSin/Cas9 causes a substantial decrease in cell viability, which was especially evident after 48 hours and 72 hours (Figure 5). For example, after 48 hours, all doses of PULSin/Cas9 caused a reduction in cell viability with the highest dose resulting in a 69% drop in viability compared to untreated cells (Figure 5a). On the contrary, EVs caused a slight increase in cell viability after 24 hours, while cell viability was identical to that of untreated cells after a 48-hour incubation period (Figure 5b). At 72 hours, the maximum dose of Cas9-loaded EVs reduced the cell viability to 74%, while the equivalent value was 36% for PULSin/Cas9 (Figure 5c). In conclusion, these results corroborate the in vitro toxicity of cationic nanoparticles and demonstrate that cell viability can be substantially improved by using EVs as a protein delivery system. 

## 3. Materials and Methods

### 3.1. Cell Lines and Culture Methods

Human MDA-MB-231 breast cancer cells (HTB-26; American Type Culture Collection (ATCC)) and murine RAW 264.7 macrophage cells (TIB-71; ATCC) were cultured in high glucose Dulbecco’s Modified Eagle Medium (DMEM) (Life Technologies, Cambridge, MA, USA) supplemented with fetal bovine serum (FBS, 10%) (Sigma, St. Louis, MO, USA), penicillin/streptomycin (1%) (Gemini Bioproducts, West Sacramento, CA, USA) and glutamine (1%) (Life Technologies, Waltham, MA, USA). All cell lines were used from passages 2 to 20 and maintained at 37 °C in 5% CO_2_. Conditioned media was obtained by seeding MDA-MB-231 cells in 150 mm dishes with DMEM supplemented with EV-depleted FBS (10%) (exosome-depleted FBS; System Biosciences, Palo Alto, CA, USA) and harvested after 48 h. Cell confluency was about 90% and cell viability was over 95% (Trypan blue). For all experiments with RAW264.7 cells, cells were maintained in complete medium supplemented with EV-depleted FBS (10%) (exosome-depleted FBS, System Biosciences, Palo Alto, CA, USA).

### 3.2. EV Isolation and Concentration by Tangential Flow Filtration

The medium harvested from MDA-MB-231 cells in culture was centrifuged (800× *g*; 30 min; Sotvall ST 16R centrifuge, Thermo Scientific, Waltham, MA, USA) to pellet down floating cells and large apoptotic bodies. Following a published protocol [45,46,65], MDA-MB-231 EVs, referred to as EVs throughout the text, were isolated using a KrosFlo Research 2i Tangential Flow Filtration System (Spectrum Labs, San Francisco, CA, USA). Briefly, MDA-MB-231 conditioned medium (0.5–0.8 L) was processed using sterile hollow fiber modified-polyethersulfone membranes designed with 0.65 μm (D02-E65U-07-S; Spectrum Labs, San Francisco, CA) and sterile hollow fiber polysulfone membranes 500 kDa (D02-S500-05-S; Spectrum Labs, San Francisco, CA, USA) molecular weight cut-off pores to remove larger components (~> 650 nm), such as cell debris and small components (~< 30 nm), such as proteins. EVs are known to be larger than 500 kDa [80] and 30 nm [81]. Filters were washed with cell-grade phosphate-buffered saline (PBS; pH 7.4; 3× area of the filter) before processing the cell culture medium. In order to keep the feed stream’s shear rate below 2000 s^−1,^ the input flow rate was set to 87 mL/minute. EVs were concentrated to approximately 50 mL and diafiltrated six times using a sucrose buffer (5% sucrose, 50 mM Tris and 2 mM MgCl; pH 7.4; 08-735B; Lonza, Alpharetta, GA) with cryoprotectant properties that was previously used in EV studies [45,46]. The final EV samples were concentrated to 6–9 mL, characterized, split into 200 µL aliquots stored at −80 °C. In order to obtain a control buffer, sucrose buffer (1 L) was diafiltered six times using a sterile filter with 500 kDa (D02-S500-05-S; Spectrum Labs, San Francisco, CA, USA) cut-off pores and divided into 200 µL aliquots stored at −80 °C. 

### 3.3. Nanoparticle Tracking Analysis

EV sample size and concentration were determined by nanoparticle tracking analysis (NTA), as previously reported [45,46,65]. EV samples were diluted (1:100 or 1:50) in cell culture grade PBS (1 mL) and measured with a NanoSight NS300 (Malvern Panalytical, Malvern, Westborough, MA, USA) (60-s measurement; 3 capture replicates).

### 3.4. Zeta Potential

EVs and nanoparticles were diluted (1:100) in sterile water (1 mL) and the zeta potential was measured by laser Doppler micro-electrophoresis (Smoluchowski’s theory) using a Zetasizer Nano ZS (ZEN 3600; Malvern Panalytical, Westborough, MA, USA). 

### 3.5. Fluorescent Labeling and Conjugation of Proteins

TrueCut Cas9 Protein v2protein (Invitrogen, Carlsbad, CA, USA) was labeled with Alexa488 microscale protein labeling kit A30006 (Thermo Fisher Scientific, Waltham, MA, USA) and PE/R-Phycoerythrin (R-PE) Conjugation Kit—Lightning-Link (ab102918) (Abcam, Cambridge, MA, USA) following the manufacturer’s instructions.

### 3.6. PULSin/Protein Nanoparticles

PULSin nanoparticles were formed using the PULSin protein transfection reagent (PolyPlus Transfection, New York, NY, USA) according to the manufacturer’s instructions, except when incubated with EVs, in which case a four times higher than suggested PULSin/protein ratio (16 µL/1 µg) was used in order to favor complexation between PULSin nanoparticles and anionic EVs. PULSin was complexed with Alexa488-labeled Cas9, Cas9-R-PE, or bovine serum albumin conjugated with Alexa Fluor 488 (Thermo Fisher Scientific, Waltham, MA, USA).

### 3.7. Lipofectamine/Protein Nanoparticles

Lipofectamine nanoparticles were formed using Lipofectamine CRISPRMAX Cas9 Transfection Reagent (Thermo Fisher Scientific, Waltham, MA, USA) according to the manufacturer’s instructions.

### 3.8. Electroporation

RAW 264.7 cells were electroporated with soluble fluorescently labeled Cas9 in solution with the Neon Transfection System (Thermo Fisher Scientific, Waltham, MA, USA) following the manufacturer’s suggested procedure for this specific cell line. 

### 3.9. Complexation of PULSin/Protein Nanoparticles with EVs

MDA-MB-231 EVs in sucrose buffer (10^10^/0.8 mL) were gently mixed by inversion with PULSin (16 µL)/protein nanoparticles (1 µg) and incubated in a water bath (37 °C) for four hours. Size-exclusion chromatography was performed to eliminate excess PULSin/Cas9, PULSin and Cas9. 

### 3.10. Size-Exclusion Chromatography

qEV original (Izon) size-exclusion chromatography columns previously washed with sucrose buffer (30 mL) were used to process 900 µL of EVs (10^10^ particles/mL), EV/PULSin/Cas9 (10^10^ particles/mL; 16 µL; 1 µg) PULSin/Cas9 (16 µL; 1 µg) and Cas9 (1 µg) following the manufacturer’s instructions. The void volume consisting of the first six eluted fractions (3 mL) was discarded. EV-rich fractions from 7 to 9 (1.5 mL) were then collected, pooled, and either analyzed or incubated with cultured cells. The concentration and size distribution profiles of EV and nanoparticle post-size-exclusion chromatography were measured by NTA.

### 3.11. Western Blot Protocol

EVs were concentrated using MilliporeSigma™ Amicon™ Ultra Centrifugal Filter Units 100 kDa molecular weight cut off (MilliporeSigma™, Temecula, CA, USA) according to the manufacturer’s instructions. After concentration, sufficient volume of EVs were taken and diluted to a final concentration of 4 × 10^10^ EVs per reaction. Briefly, centrifugation was performed at 4,000 × *g* for 30 min at 4 °C. The retentate was collected and complexed with PULSin/Cas9. Size-exclusion chromatography was performed on 500–600 µL of EVs (4 × 10^10^ particles total), EV/PULSin/Cas9 (4 × 10^10^ particles; 64 µL PULSin; 4 µg Cas9) PULSin/Cas9 (64 µL; 4 µg), Cas9 (4 µg) as described above. Size-exclusion chromatography-processed EVs, EV/PULSin/Cas9, PULSin/Cas9, and Cas9 samples were combined with sodium dodecyl sulfate (SDS)-sample buffer 6× reducing (Boston Bioproducts) and heated (five minutes; 95 °C). MDA-MB-231 cell homogenate was used as a control. In order to obtain the cell homogenates, radioimmunoprecipitation assay (RIPA) buffer (Thermo Fisher Scientific, Waltham, MA, USA), with cOmplete^TM^ Protease Inhibitor Cocktail (Roche), was added to the culture plates kept on ice for five to seven minutes. Cells were then scraped, collected into low protein binding microtubes and centrifuged (12,000 × *g* for five minutes). The supernatant was further processed for analysis. All samples were normalized according to the volume of the size-exclusion chromatography elution fractions 7–9, except cellular homogenate, which was normalized to the protein concentration of the EV sample (~0.6 μg/well), as determined by bicinchoninic acid (BCA) assay (Thermo Fisher Scientific, Waltham, MA, USA). Samples and Cas9 standards were loaded, electrophoresed on a polyacrylamide gel (4–12%) and analyzed by Western blot. The following antibodies were used: mouse monoclonal anti-cluster of differentiation (CD)81 (1:500 dilution; clone (B11): sc-166029; Santa Cruz, Dallas, TX, USA), mouse monoclonal anti-CD9 (1:500 dilution; 10626D; Thermo Fisher Scientific, Waltham, MA, USA), rabbit polyclonal anti Calnexin (1:500 dilution; clone GTX112886; GeneTex, Irvine, CA, USA), recombinant, rabbit monoclonal anti CD63 (1:500 dilution; ab134045; Abcam, Cambridge, MA, USA), rabbit polyclonal anti-heat shock cognate 71 (Hsc70) (1:200 dilution; ab1427; Abcam, Cambridge, MA, USA), rabbit polyclonal anti-annexin V (1:500 dilution; ab14196; Abcam, Cambridge, MA, USA), rabbit polyclonal anti-Cas9 (1:500 dilution; ABS2202-25UG; Millipore Sigma, Temecula, CA, USA), anti-rabbit immunoglobulin G (IgG) secondary horseradish peroxidase (HRP)-linked antibody (1:3000; Cell Signaling Technology, Danvers, MA, USA) and anti-mouse IgG secondary HRP-linked antibody (1:3000; Thermo Fisher Scientific, Waltham, MA, USA). A protein ladder, the ECL™ Rainbow™ Marker—Full range (Amersham, Chicago, IL, USA), was also used. The immunoreactive bands were detected with the SuperSignal West Femto detection kit (Thermo Fisher Scientific, Waltham, MA, USA). 

### 3.12. Immunofluorescence Staining

RAW264.7 cells were grown on µ-Slide 8 Well ibiTreat chamber slides (Ibidi). When cells were 80–90% confluent (10^4^ cells/well), they were incubated with a fixed-dose (0.2 µg of Cas9 protein) of Cas9-loaded EVs (EV/PULSin/Cas9) (10^3^ EVs/cell) for three hours, Cas9-loaded PULSin (PULSin/Cas9) (1 µL) for four hours according to the manufacturer’s instructions, or Cas9-loaded Lipofectamine CRISPRMAX Cas9 Transfection Reagent (0.3 µL) for 24 h, according to the manufacturer’s instructions. Cells were separately electroporated to promote Cas9 intracellular delivery and then incubated with fresh media for 24 h. After cell medium removal, cells were washed once with sterile PBS (pH 7.4), fixed using a formaldehyde solution (4%) (Electron Microscopy Sciences, Hatfield, PA) for 20 min at room temperature and washed three more times with sterile PBS (pH 7.4) to remove the remaining fixative. Cells were then incubated with glycine (0.1 M) and washed three times with sterile PBS (pH 7.4) in order to remove excess amino acid solution. After fixation, cells were stained with Hoechst for 15 min. Confocal imaging of the cell monolayers was performed using a Zeiss LSM 880 laser scanning microscope with 40× and 63× oil objectives. Images were processed using the Zen software (Zeiss, Jena, Germany).

### 3.13. Flow Cytometry

MDA-MB-231 cells (1 × 10^5^ cells/well in 24-well plates) were incubated with protein-loaded MDA-MB-231 cell-derived EVs (post-size-exclusion chromatography; 10^3^ EVs/cell) for three hours in EV-free medium. RAW 264.7 cells (1 × 10^5^ cells/well in 24-well plates) were incubated with either two different doses (post-size-exclusion chromatography; 10^3^ EVs/cell and 10^4^ EVs/cell) or four different doses of protein-loaded MDA-MB-231 cell-derived EVs (post-size-exclusion chromatography; 2 × 10^4^, 4 × 10^3^, 2 × 10^3^, or 2 × 10^2^ EVs/cell) for three hours in EV-free medium. Cells were separately incubated with four different doses (1, 0.2, 0.1, or 0.01 µg of protein/10^5^ cells) of PULSin/protein nanoparticles, or Lipofectamine/protein nanoparticles complexed following the manufacturer’s instructions. Cells were also electroporated with four different doses of Cas9 (1, 0.2, 0.1, or 0.01 µg/10^5^ cells) to promote protein intracellular delivery. Based on our previous studies, the delivery efficiency of EVs reaches a saturation point at 2 × 10^4^ EVs/cell [45,65], which was, therefore, used as the highest dose in this study. The maximum EV dose was then decreased 5, 10 and 100 times to assess dose-responses. The EV doses were compared to those of the maximum suggested quantity of protein/10^5^ cells, administered by PULSin/protein nanoparticles, Lipofectamine/protein nanoparticles, and electroporation. The initial dose (1 µg of protein/10^5^ cells) was decreased following the same ratios chosen for EVs/cell, namely 1/5, 1/10, and 1/100, 15 min prior to the flow cytometry analysis, Hoechst 33258 pentahydrate (bisbenzimide) (2 μL; Invitrogen, Carlsbad, CA, USA) was added to each well. RAW 264.7 cells were washed once with sterile PBS, trypsinized, pelleted (800× *g* for five minutes), resuspended in PBS (200 μL). RAW cells in solution were stained with Sytox Red (Thermo Fisher Scientific, Waltham, MA) for 20 min at room temperature and immediately analyzed by flow cytometry (Attune NxT flow cytometer; Thermo Fisher Scientific, Waltham, MA, USA). A comparable number of cells (≥1.5 × 10^4^) was analyzed for each sample and the data from the Sytox Red-negative, Hoechst-positive and Alexa488-positive populations were recorded. 

### 3.14. MTS (3-(4,5-dimethylthiazol-2-yl)-5-(3-carboxymethoxyphenyl)-2-(4-sulfophenyl)-2H-tetrazolium) Viability Assay

RAW 264.7 cells were cultured in 96-well plates (10^3^ cells/well). When cells were 80–90% confluent, they were incubated with various doses of protein-loaded EVs (2 × 10^4^, 4 × 10^3^, 2 × 10^3^ and 2 × 10^2^ EVs/cell) or protein-loaded PULSin (1, 0.2, 0.1, or 0.01 µg/10^5^ cells) for 4, 24, or 48 h. MTS reagent (Abcam, Cambridge, MA) (10% volume) was added to each well and cells were incubated for one hour at 37 °C and at 5% of CO_2_. The sample absorbance at 490 nm was measured using a plate reader (Synergy HT; BioTek, Winooski, VT, USA).

### 3.15. Statistical Analysis

The statistical tests used to analyze the data shown in each figure are indicated in the caption. Graph construction and statistical analyses were performed using Prism 7.0a software (GraphPad).

## 4. Conclusions

Proteins are important therapeutic agents for many applications, including genome editing, for example, Cas9 [66] and enzyme replacement therapy [82]. However, protein delivery is challenging as proteins are sensitive to structural alterations, can be recognized as foreign by the immune system, are easily degraded by proteases and have limited intracellular uptake [83]. To overcome such challenges, various synthetic nanoparticles and EV loading methods have been used for protein delivery. These methods often display several disadvantages, including cytotoxicity and damage of proteins and EV membranes [33,34,59,77], necessitating the development of improved methods.

In this study, a hybrid approach consisting of the complexation of synthetic nanoparticles with EVs was developed for protein loading. The results demonstrate that EV-mediated delivery is compatible with various molecular weight proteins (66–400 kDa) and results in similar uptake efficiencies as commercial protein transfection reagents and improved uptake compared to electroporation. Additionally, cell viability was improved with EV-based delivery compared to conventional cationic transfection reagents. It is likely that the developed method will have to be further optimized (for example, ratio of synthetic lipids to EVs) depending on the EV type, as there is considerable heterogeneity in terms of EV sources and isolation methods, which could impact protein complexation and cellular uptake. Furthermore, the results were obtained with macrophages, which exhibit high levels of endocytosis [70], which is the primary pathway of EV internalization [71]. Therefore, extension of EV incubation times (beyond three hours) may be necessary for recipient cells that exhibit lower levels of endocytosis. 

An additional consideration that should be taken into account is the possibility of harmful endogenous cargo molecules, such as potential immortalization agents, in EVs derived from cancer cell lines [13]. Such agents could potentially exhibit tumorigenicity or lead to accelerated growth of existing tumors. Additionally, EVs from cancer cells may display abnormal interactions with circulating components [65]. Despite the potential safety concerns of cancer cell-derived EVs, several initial clinical trials have demonstrated that such EVs have acceptable safety profiles [79]. The endogenous cargo of cancer cell-derived EVs can also have beneficial effects when used for cancer immunotherapy [84] and could contribute in an additive or synergistic manner to the activity of loaded therapeutic agents. An improved understanding of the effects of cancer-derived EVs on the activation of the host immune system could be leveraged with protein loading protocols for the development of anti-cancer vaccines. Additionally, other cells, such as mesenchymal stem cells, have been reported to secrete EVs with beneficial endogenous cargo molecules that mediate anti-inflammatory, anti-fibrotic, anti-apoptotic, and pro-angiogenic responses [74,85]. Overall, the simple, quick and versatile method described in this study is likely to open up opportunities for rapid exploration of EVs for protein delivery. 

## Data Availability

Data are available in the main manuscript and from the corresponding authors upon reasonable request.

## Supplementary Materials

The following are available online at https://www.mdpi.com/article/10.3390/ph14040356/s1, Figure S1: Samples size distribution profiles.

## References

[1] Raposo G., Stoorvogel W. (2013). Extracellular vesicles: Exosomes, microvesicles, and friends. J. Cell Biol..

[2] Yanez-Mo M., Siljander P.R., Andreu Z., Zavec A.B., Borras F.E., Buzas E.I., Buzas K., Casal E., Cappello F., Carvalho J. (2015). Biological properties of extracellular vesicles and their physiological functions. J. Extracell. Vesicles.

[3] Van Niel G., D’Angelo G., Raposo G. (2018). Shedding light on the cell biology of extracellular vesicles. Nat. Rev. Mol. Cell Biol..

[4] Hu T., Wolfram J., Srivastava S. (2020). Extracellular Vesicles in Cancer Detection: Hopes and Hypes. Trends Cancer.

[5] Chen S., Datta-Chaudhuri A., Deme P., Dickens A., Dastgheyb R., Bhargava P., Bi H., Haughey N.J. (2019). Lipidomic characterization of extracellular vesicles in human serum. J. Circ. Biomark..

[6] Bastos-Amador P., Royo F., Gonzalez E., Conde-Vancells J., Palomo-Diez L., Borras F.E., Falcon-Perez J.M. (2012). Proteomic analysis of microvesicles from plasma of healthy donors reveals high individual variability. J. Proteom..

[7] Walker S.A., Aguilar Diaz De Leon J.S., Busatto S., Wurtz G.A., Zubair A.C., Borges C.R., Wolfram J. (2020). Glycan Node Analysis of Plasma-Derived Extracellular Vesicles. Cells.

[8] Hunter M.P., Ismail N., Zhang X., Aguda B.D., Lee E.J., Yu L., Xiao T., Schafer J., Lee M.L., Schmittgen T.D. (2008). Detection of microRNA expression in human peripheral blood microvesicles. PLoS ONE.

[9] Thery C., Zitvogel L., Amigorena S. (2002). Exosomes: Composition, biogenesis and function. Nat. Rev. Immunol..

[10] Hauser P., Wang S., Didenko V.V. (2017). Apoptotic Bodies: Selective Detection in Extracellular Vesicles. Methods Mol. Biol..

[11] Andaloussi S.E., Mager I., Breakefield X.O., Wood M.J. (2013). Extracellular vesicles: Biology and emerging therapeutic opportunities. Nat. Rev. Drug Discov..

[12] Thery C., Witwer K.W., Aikawa E., Alcaraz M.J., Anderson J.D., Andriantsitohaina R., Antoniou A., Arab T., Archer F., Atkin-Smith G.K. (2018). Minimal information for studies of extracellular vesicles 2018 (MISEV2018): A position statement of the International Society for Extracellular Vesicles and update of the MISEV2014 guidelines. J. Extracell. Vesicles.

[13] Witwer K.W., Wolfram J. (2021). Extracellular vesicles versus synthetic nanoparticles for drug delivery. Nat. Rev. Mater..

[14] Batrakova E.V., Kim M.S. (2015). Using exosomes, naturally-equipped nanocarriers, for drug delivery. J. Control. Release.

[15] Bari E., Ferrarotti I., Di Silvestre D., Grisoli P., Barzon V., Balderacchi A., Torre M.L., Rossi R., Mauri P., Corsico A.G. (2019). Adipose Mesenchymal Extracellular Vesicles as Alpha-1-Antitrypsin Physiological Delivery Systems for Lung Regeneration. Cells.

[16] Gentile E., Cilurzo F., Di Marzio L., Carafa M., Ventura C.A., Wolfram J., Paolino D., Celia C. (2013). Liposomal chemotherapeutics. Future Oncol..

[17] Wolfram J., Ferrari M. (2019). Clinical Cancer Nanomedicine. Nano Today.

[18] Khalid A., Persano S., Shen H., Zhao Y., Blanco E., Ferrari M., Wolfram J. (2017). Strategies for improving drug delivery: Nanocarriers and microenvironmental priming. Expert Opin. Drug Deliv..

[19] Busatto S., Pham A., Suh A., Shapiro S., Wolfram J. (2019). Organotropic drug delivery: Synthetic nanoparticles and extracellular vesicles. Biomed. Microdevices.

[20] Wolfram J., Zhu M., Yang Y., Shen J., Gentile E., Paolino D., Fresta M., Nie G., Chen C., Shen H. (2015). Safety of Nanoparticles in Medicine. Curr. Drug Targets.

[21] Busatto S., Walker S.A., Grayson W., Pham A., Tian M., Nesto N., Barklund J., Wolfram J. (2020). Lipoprotein-based drug delivery. Adv. Drug Deliv Rev..

[22] Scavo M.P., Gentile E., Wolfram J., Gu J., Barone M., Evangelopoulos M., Martinez J.O., Liu X., Celia C., Tasciotti E. (2015). Multistage vector delivery of sulindac and silymarin for prevention of colon cancer. Colloids Surf. B Biointerfaces.

[23] Shen J., Wu X., Lee Y., Wolfram J., Yang Z., Mao Z.W., Ferrari M., Shen H. (2015). Porous silicon microparticles for delivery of siRNA therapeutics. J. Vis. Exp..

[24] Shen J., Liu H., Mu C., Wolfram J., Zhang W., Kim H.C., Zhu G., Hu Z., Ji L.N., Liu X. (2017). Multi-step encapsulation of chemotherapy and gene silencing agents in functionalized mesoporous silica nanoparticles. Nanoscale.

[25] Mi Y., Mu C., Wolfram J., Deng Z., Hu T.Y., Liu X., Blanco E., Shen H., Ferrari M. (2016). A Micro/Nano Composite for Combination Treatment of Melanoma Lung Metastasis. Adv. Healthc. Mater..

[26] Martins J.P., das Neves J., de la Fuente M., Celia C., Florindo H., Gunday-Tureli N., Popat A., Santos J.L., Sousa F., Schmid R. (2020). The solid progress of nanomedicine. Drug Deliv. Transl. Res..

[27] Reshke R., Taylor J.A., Savard A., Guo H., Rhym L.H., Kowalski P.S., Trung M.T., Campbell C., Little W., Anderson D.G. (2020). Reduction of the therapeutic dose of silencing RNA by packaging it in extracellular vesicles via a pre-microRNA backbone. Nat. Biomed. Eng..

[28] Zhang S., Zhao B., Jiang H., Wang B., Ma B. (2007). Cationic lipids and polymers mediated vectors for delivery of siRNA. J. Control. Release.

[29] Scott B., Shen J., Nizzero S., Boom K., Persano S., Mi Y., Liu X., Zhao Y., Blanco E., Shen H. (2016). A pyruvate decarboxylase-mediated therapeutic strategy for mimicking yeast metabolism in cancer cells. Pharmacol. Res..

[30] Weiss A., Neuberg P., Philippot S., Erbacher P., Weill C.O. (2011). Intracellular peptide delivery using amphiphilic lipid-based formulations. Biotechnol. Bioeng..

[31] Lv H., Zhang S., Wang B., Cui S., Yan J. (2006). Toxicity of cationic lipids and cationic polymers in gene delivery. J. Control. Release.

[32] Toy R., Pradhan P., Ramesh V., Di Paolo N.C., Lash B., Liu J., Blanchard E.L., Pinelli C.J., Santangelo P.J., Shayakhmetov D.M. (2019). Modification of primary amines to higher order amines reduces in vivo hematological and immunotoxicity of cationic nanocarriers through TLR4 and complement pathways. Biomaterials.

[33] Soenen S.J., Brisson A.R., De Cuyper M. (2009). Addressing the problem of cationic lipid-mediated toxicity: The magnetoliposome model. Biomaterials.

[34] Knudsen K.B., Northeved H., Kumar P.E., Permin A., Gjetting T., Andresen T.L., Larsen S., Wegener K.M., Lykkesfeldt J., Jantzen K. (2015). In vivo toxicity of cationic micelles and liposomes. Nanomed. Nanotechnol. Biol. Med..

[35] Walker S., Busatto S., Pham A., Tian M., Suh A., Carson K., Quintero A., Lafrence M., Malik H., Santana M.X. (2019). Extracellular vesicle-based drug delivery systems for cancer treatment. Theranostics.

[36] Aspe J.R., Osterman C.J.D., Jutzy J.M., Deshields S., Whang S., Wall N.R. (2014). Enhancement of Gemcitabine sensitivity in pancreatic adenocarcinoma by novel exosome-mediated delivery of the Survivin-T34A mutant. J. Extracell. Vesicles.

[37] Lattanzi L., Federico M. (2012). A strategy of antigen incorporation into exosomes: Comparing cross-presentation levels of antigens delivered by engineered exosomes and by lentiviral virus-like particles. Vaccine.

[38] Gee P., Lung M.S.Y., Okuzaki Y., Sasakawa N., Iguchi T., Makita Y., Hozumi H., Miura Y., Yang L.F., Iwasaki M. (2020). Extracellular nanovesicles for packaging of CRISPR-Cas9 protein and sgRNA to induce therapeutic exon skipping. Nat. Commun..

[39] Campbell L.A., Coke L.M., Richie C.T., Fortuno L.V., Park A.Y., Harvey B.K. (2019). Gesicle-Mediated Delivery of CRISPR/Cas9 Ribonucleoprotein Complex for Inactivating the HIV Provirus. Mol. Ther..

[40] Mangeot P.E., Risson V., Fusil F., Marnef A., Laurent E., Blin J., Mournetas V., Massourides E., Sohier T.J.M., Corbin A. (2019). Genome editing in primary cells and in vivo using viral-derived Nanoblades loaded with Cas9-sgRNA ribonucleoproteins. Nat. Commun..

[41] Guo S.C., Tao S.C., Yin W.J., Qi X., Yuan T., Zhang C.Q. (2017). Exosomes derived from platelet-rich plasma promote the re-epithelization of chronic cutaneous wounds via activation of YAP in a diabetic rat model. Theranostics.

[42] Tao S.C., Yuan T., Rui B.Y., Zhu Z.Z., Guo S.C., Zhang C.Q. (2017). Exosomes derived from human platelet-rich plasma prevent apoptosis induced by glucocorticoid-associated endoplasmic reticulum stress in rat osteonecrosis of the femoral head via the Akt/Bad/Bcl-2 signal pathway. Theranostics.

[43] Hu Y., Rao S.S., Wang Z.X., Cao J., Tan Y.J., Luo J., Li H.M., Zhang W.S., Chen C.Y., Xie H. (2018). Exosomes from human umbilical cord blood accelerate cutaneous wound healing through miR-21-3p-mediated promotion of angiogenesis and fibroblast function. Theranostics.

[44] Abel F., Murke F., Gaida M., Garnier N., Ochsenfarth C., Theiss C., Thielmann M., Kleinbongard P., Giebel B., Peters J. (2020). Extracellular vesicles isolated from patients undergoing remote ischemic preconditioning decrease hypoxia-evoked apoptosis of cardiomyoblasts after isoflurane but not propofol exposure. PLoS ONE.

[45] Tian M., Ticer T., Wang Q., Walker S., Pham A., Suh A., Busatto S., Davidovich I., Al-Kharboosh R., Lewis-Tuffin L. (2020). Adipose-Derived Biogenic Nanoparticles for Suppression of Inflammation. Small.

[46] Busatto S., Vilanilam G., Ticer T., Lin W.L., Dickson D.W., Shapiro S., Bergese P., Wolfram J. (2018). Tangential Flow Filtration for Highly Efficient Concentration of Extracellular Vesicles from Large Volumes of Fluid. Cells.

[47] Bellei B., Migliano E., Tedesco M., Caputo S., Papaccio F., Lopez G., Picardo M. (2018). Adipose tissue-derived extracellular fraction characterization: Biological and clinical considerations in regenerative medicine. Stem Cell Res. Ther..

[48] Wang X. (2017). Isolation of Extracellular Vesicles from Breast Milk. Methods Mol. Biol..

[49] Pisano C., Galley J., Elbahrawy M., Wang Y., Farrell A., Brigstock D., Besner G.E. (2020). Human Breast Milk-Derived Extracellular Vesicles in the Protection Against Experimental Necrotizing Enterocolitis. J. Pediatr. Surg..

[50] Zhou Q., Li M., Wang X., Li Q., Wang T., Zhu Q., Zhou X., Wang X., Gao X., Li X. (2012). Immune-related microRNAs are abundant in breast milk exosomes. Int. J. Biol. Sci..

[51] Lee R., Ko H.J., Kim K., Sohn Y., Min S.Y., Kim J.A., Na D., Yeon J.H. (2020). Anti-melanogenic effects of extracellular vesicles derived from plant leaves and stems in mouse melanoma cells and human healthy skin. J. Extracell. Vesicles.

[52] Cao M., Yan H., Han X., Weng L., Wei Q., Sun X., Lu W., Wei Q., Ye J., Cai X. (2019). Ginseng-derived nanoparticles alter macrophage polarization to inhibit melanoma growth. J. Immunother. Cancer.

[53] Matsuda A., Moirangthem A., Angom R.S., Ishiguro K., Driscoll J., Yan I.K., Mukhopadhyay D., Patel T. (2020). Safety of bovine milk derived extracellular vesicles used for delivery of RNA therapeutics in zebrafish and mice. J. Appl. Toxicol..

[54] Izumi H., Tsuda M., Sato Y., Kosaka N., Ochiya T., Iwamoto H., Namba K., Takeda Y. (2015). Bovine milk exosomes contain microRNA and mRNA and are taken up by human macrophages. J. Dairy Sci..

[55] Wolf T., Baier S.R., Zempleni J. (2015). The Intestinal Transport of Bovine Milk Exosomes Is Mediated by Endocytosis in Human Colon Carcinoma Caco-2 Cells and Rat Small Intestinal IEC-6 Cells. J. Nutr..

[56] Munagala R., Aqil F., Jeyabalan J., Gupta R.C. (2016). Bovine milk-derived exosomes for drug delivery. Cancer Lett..

[57] Baier S.R., Nguyen C., Xie F., Wood J.R., Zempleni J. (2014). MicroRNAs are absorbed in biologically meaningful amounts from nutritionally relevant doses of cow milk and affect gene expression in peripheral blood mononuclear cells, HEK-293 kidney cell cultures, and mouse livers. J. Nutr..

[58] Iannotta D., Yang M., Celia C., Di Marzio L., Wolfram J. (2021). Extracellular vesicle therapeutics from plasma and adipose tissue. Nano Today.

[59] Haney M.J., Klyachko N.L., Zhao Y., Gupta R., Plotnikova E.G., He Z., Patel T., Piroyan A., Sokolsky M., Kabanov A.V. (2015). Exosomes as drug delivery vehicles for Parkinson’s disease therapy. J. Control. Release.

[60] Kooijmans S.A.A., Stremersch S., Braeckmans K., de Smedt S.C., Hendrix A., Wood M.J.A., Schiffelers R.M., Raemdonck K., Vader P. (2013). Electroporation-induced siRNA precipitation obscures the efficiency of siRNA loading into extracellular vesicles. J. Control. Release.

[61] Lin Y., Wu J., Gu W., Huang Y., Tong Z., Huang L., Tan J. (2018). Exosome-Liposome Hybrid Nanoparticles Deliver CRISPR/Cas9 System in MSCs. Adv. Sci..

[62] Shtam T.A., Kovalev R.A., Varfolomeeva E.Y., Makarov E.M., Kil Y.V., Filatov M.V. (2013). Exosomes are natural carriers of exogenous siRNA to human cells in vitro. Cell Commun. Signal..

[63] Fukuhara T., Tani H., Shiokawa M., Goto Y., Abe T., Taketomi A., Shirabe K., Maehara Y., Matsuura Y. (2011). Intracellular delivery of serum-derived hepatitis C virus. Microbes Infect..

[64] Marschall A.L., Zhang C., Frenzel A., Schirrmann T., Hust M., Perez F., Dubel S. (2014). Delivery of antibodies to the cytosol: Debunking the myths. MAbs.

[65] Busatto S., Yang Y., Walker S.A., Davidovich I., Lin W.H., Lewis-Tuffin L., Anastasiadis P.Z., Sarkaria J., Talmon Y., Wurtz G. (2020). Brain metastases-derived extracellular vesicles induce binding and aggregation of low-density lipoprotein. J. Nanobiotechnology.

[66] Adli M. (2018). The CRISPR tool kit for genome editing and beyond. Nat. Commun..

[67] Xiao F., Nicholson C., Hrabe J., Hrabetova S. (2008). Diffusion of flexible random-coil dextran polymers measured in anisotropic brain extracellular space by integrative optical imaging. Biophys. J..

[68] Chen F., Alphonse M., Liu Q. (2020). Strategies for nonviral nanoparticle-based delivery of CRISPR/Cas9 therapeutics. Wiley Interdiscip. Rev. Nanomed. Nanobiotechnology.

[69] Mittal R., Sharma R., Raghavarao K. (2019). Aqueous two-phase extraction of R-Phycoerythrin from marine macro-algae, Gelidium pusillum. Bioresour. Technol..

[70] Samuelsson E., Shen H., Blanco E., Ferrari M., Wolfram J. (2017). Contribution of Kupffer cells to liposome accumulation in the liver. Colloids Surf. B. Biointerfaces.

[71] Costa Verdera H., Gitz-Francois J.J., Schiffelers R.M., Vader P. (2017). Cellular uptake of extracellular vesicles is mediated by clathrin-independent endocytosis and macropinocytosis. J. Control. Release.

[72] Sokolova V., Kozlova D., Knuschke T., Buer J., Westendorf A.M., Epple M. (2013). Mechanism of the uptake of cationic and anionic calcium phosphate nanoparticles by cells. Acta Biomater..

[73] Lin J., Alexander-Katz A. (2013). Cell membranes open “doors” for cationic nanoparticles/biomolecules: Insights into uptake kinetics. ACS Nano.

[74] Ali M., Pham A., Wang X., Wolfram J., Pham S. (2020). Extracellular vesicles for treatment of solid organ ischemia-reperfusion injury. Am. J. Transplant..

[75] Borrelli D.A., Yankson K., Shukla N., Vilanilam G., Ticer T., Wolfram J. (2018). Extracellular vesicle therapeutics for liver disease. J. Control. Release.

[76] Choi S.O., Kim Y.C., Lee J.W., Park J.H., Prausnitz M.R., Allen M.G. (2012). Intracellular protein delivery and gene transfection by electroporation using a microneedle electrode array. Small.

[77] Weaver J.C. (1993). Electroporation: A general phenomenon for manipulating cells and tissues. J. Cell. Biochem..

[78] Klyachko N.L., Arzt C.J., Li S.M., Gololobova O.A., Batrakova E.V. (2020). Extracellular Vesicle-Based Therapeutics: Preclinical and Clinical Investigations. Pharmaceutics.

[79] Elsharkasy O.M., Nordin J.Z., Hagey D.W., de Jong O.G., Schiffelers R.M., Andaloussi S.E., Vader P. (2020). Extracellular vesicles as drug delivery systems: Why and how?. Adv. Drug Deliv. Rev..

[80] Corso G., Mager I., Lee Y., Gorgens A., Bultema J., Giebel B., Wood M.J.A., Nordin J.Z., Andaloussi S.E. (2017). Reproducible and scalable purification of extracellular vesicles using combined bind-elute and size exclusion chromatography. Sci. Rep..

[81] Murphy D.E., de Jong O.G., Brouwer M., Wood M.J., Lavieu G., Schiffelers R.M., Vader P. (2019). Extracellular vesicle-based therapeutics: Natural versus engineered targeting and trafficking. Exp. Mol. Med..

[82] Oder D., Nordbeck P., Wanner C. (2016). Long Term Treatment with Enzyme Replacement Therapy in Patients with Fabry Disease. Nephron.

[83] Yin L., Yuvienco C., Montclare J.K. (2017). Protein based therapeutic delivery agents: Contemporary developments and challenges. Biomaterials.

[84] Taghikhani A., Farzaneh F., Sharifzad F., Mardpour S., Ebrahimi M., Hassan Z.M. (2020). Engineered Tumor-Derived Extracellular Vesicles: Potentials in Cancer Immunotherapy. Front. Immunol..

[85] Suh A., Pham A., Cress M.J., Pincelli T., TerKonda S.P., Bruce A.J., Zubair A.C., Wolfram J., Shapiro S.A. (2019). Adipose-derived cellular and cell-derived regenerative therapies in dermatology and aesthetic rejuvenation. Ageing Res. Rev..

