# Supplementary material for: A Simple and Quick Method for Loading Proteins in Extracellular Vesicles
Source: Pharmaceuticals (Basel). 2021 Apr 13;14(4):356. doi: 10.3390/ph14040356 (PMC8069621; doi:10.3390/ph14040356)
Supplement: Supplementary file 1 [file pharmaceuticals-14-00356-s001.pdf]

Supplementary Material

A simple and quick method for loading proteins in extracellular vesicles

Sara Busatto [1,2,3,*], Dalila Iannotta [1,4], Sierra A. Walker[1], Luisa Di Marzio[4], and Joy Wolfram [1,5,*]

[1] Department of Biochemistry and Molecular Biology, Mayo Clinic, Jacksonville, FL, United States
[2] Vascular Biology Program, Boston Children's Hospital, Boston, MA, United States
[3] Department of Surgery, Boston Children's Hospital, and Harvard Medical School, Boston, MA, United States
[4] Department of Pharmacy, University of Chieti – Pescara "G. d'Annunzio", Chieti, Italy
[5] Department of Nanomedicine, Houston Methodist Research Institute, Houston TX, USA

* Correspondence: sara.busatto@childrens.harvard.edu and wolfram.joy@mayo.edu

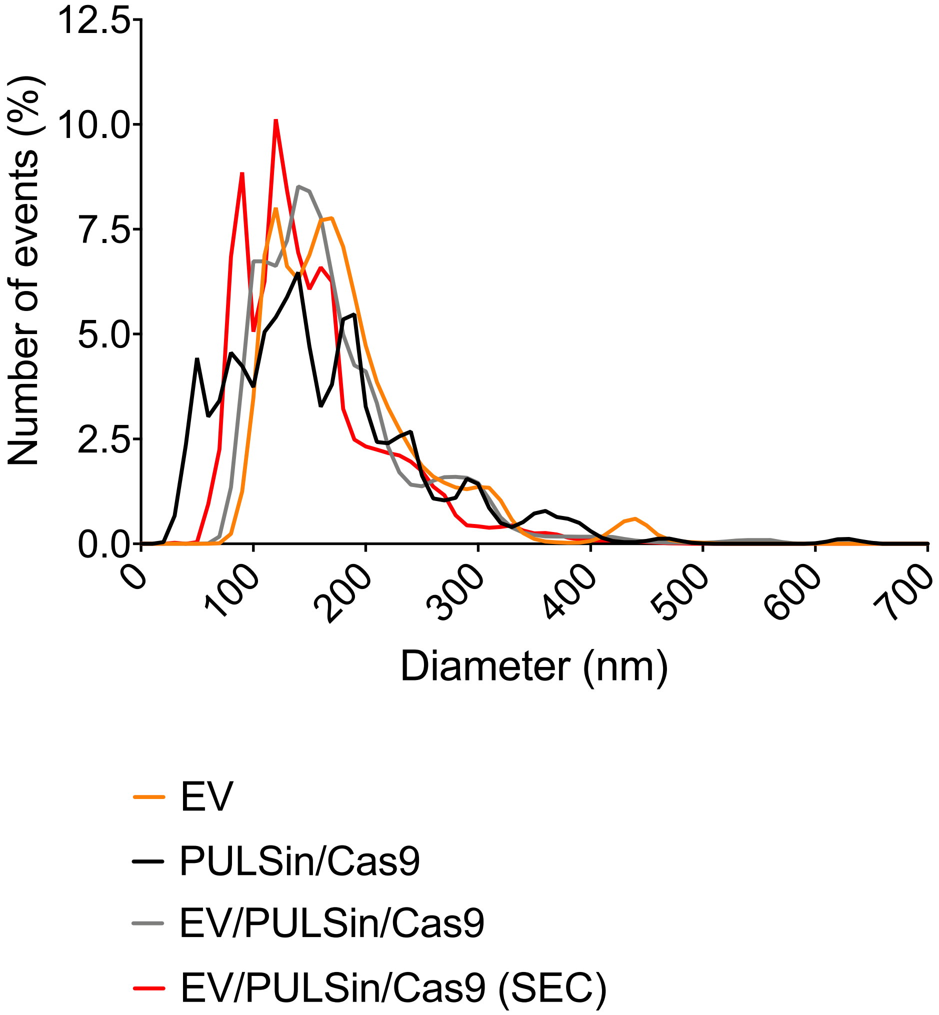

Supplementary Figure 1. Samples size distribution profiles (10 nm increments) determined by nanoparticle tracking analysis (data are presented as the mean of three biological replicates in percent of the total). Cas9, CRISPR associated protein 9; EV, extracellular vesicles; SEC, size exclusion chromatography.
